# Evaluating the Usability and Quality of a Clinical Mobile App for Assisting Physicians in Head Computed Tomography Scan Ordering: Mixed Methods Study

**DOI:** 10.2196/55790

**Published:** 2024-09-09

**Authors:** Zahra Meidani, Aydine Omidvar, Hossein Akbari, Fatemeh Asghari, Reza Khajouei, Zahra Nazemi, Ehsan Nabovati, Felix Holl

**Affiliations:** 1 Health Information Management Research Center Kashan University of Medical Sciences Kashan Iran; 2 Department of Neurosurgery Kashan University of Medical Sciences Kashan Iran; 3 Department of Epidemiology & Biostatistics Kashan University of Medical Sciences Kashan Iran; 4 Department of Health Information Sciences, Faculty of Management and Medical Information Sciences Kerman University of Medical Sciences Kerman Iran; 5 DigiHealth Institute Neu-Ulm University of Applied Sciences Neu-Ulm Germany

**Keywords:** mobile apps, user-centered design, user-computer interface, physicians, tomography, x-ray computed, mobile phone

## Abstract

**Background:**

Among the numerous factors contributing to health care providers’ engagement with mobile apps, including user characteristics (eg, dexterity, anatomy, and attitude) and mobile features (eg, screen and button size), usability and quality of apps have been introduced as the most influential factors.

**Objective:**

This study aims to investigate the usability and quality of the Head Computed Tomography Scan Appropriateness Criteria (HAC) mobile app for physicians’ computed tomography scan ordering.

**Methods:**

Our study design was primarily based on methodological triangulation by using mixed methods research involving quantitative and qualitative think-aloud usability testing, quantitative analysis of the Mobile Apps Rating Scale (MARS) for quality assessment, and debriefing across 3 phases. In total, 16 medical interns participated in quality assessment and testing usability characteristics, including efficiency, effectiveness, learnability, errors, and satisfaction with the HAC app.

**Results:**

The efficiency and effectiveness of the HAC app were deemed satisfactory, with ratings of 97.8% and 96.9%, respectively. MARS assessment scale indicated the overall favorable quality score of the HAC app (82 out of 100). Scoring 4 MARS subscales, Information (73.37 out of 100) and Engagement (73.48 out of 100) had the lowest scores, while Aesthetics had the highest score (87.86 out of 100). Analysis of the items in each MARS subscale revealed that in the Engagement subscale, the lowest score of the HAC app was “customization” (63.6 out of 100). In the Functionality subscale, the HAC app’s lowest value was “performance” (67.4 out of 100). Qualitative think-aloud usability testing of the HAC app found notable usability issues grouped into 8 main categories: lack of finger-friendly touch targets, poor search capabilities, input problems, inefficient data presentation and information control, unclear control and confirmation, lack of predictive capabilities, poor assistance and support, and unclear navigation logic.

**Conclusions:**

Evaluating the quality and usability of mobile apps using a mixed methods approach provides valuable information about their functionality and disadvantages. It is highly recommended to embrace a more holistic and mixed methods strategy when evaluating mobile apps, because results from a single method imperfectly reflect trustworthy and reliable information regarding the usability and quality of apps.

## Introduction

### Background

Mobile devices and mobile health (mHealth) apps have equipped the health care system with a strategy to improve health through enhanced self-management among patients and access to educational materials for health care professionals [1]. Considering their advantages regarding the fastest and most convenient ways to access health care services, they have been introduced as effective eHealth technology to address health priorities [2]. Recently, a global initiative has been launched to apply mobile technologies to provide health care services and manage various diseases [3]. A 2015 World Health Organization survey revealed that 15,000 mobile apps were available for health care use [4]. However, the continuity in the use of apps is highly challenging, and existing evidence presented poor user engagement and relatively high drop-out rates on apps among patients and health care providers (HCPs) [5]. Earlier research revealed that nearly half of mHealth app users avoid continuous use of them [6]. The drop-out rates in app-based interventions for chronic diseases were reported to be 43% (95% CI 29%-57%) in a meta-analysis by Meyerowitz-Katz et al [7].

Usability has been introduced as a surrogate marker for app quality and user engagement with them to address this challenge [8-10]. Given the significance, assessing the usability and quality of mobile apps occupies a crucial part of app development and users’ overall assessment of app quality [6,8]. However, emerging research has debated that mobile apps suffer from usability and quality issues and are limited by their ability to address users’ needs [9,11,12]. Physicians use many mobile apps to access a wide range of knowledge and information in educational materials, drug reference guides, x-ray results, laboratory test information, and clinical guidelines [13]. Medical apps were positively perceived, with physicians reporting increased dependency on the apps. The use of apps in the medical setting has steadily grown in recent years [14]. While a considerable number of physicians now use mobile devices and apps for clinical practices globally [7], there are also reports of drop-out rate and short-term engagement among physicians with these mobile apps [1]. Arguably, no clear understanding exists of the physicians’ motivations and interests in adopting and long-term use of mobile apps [8]. A variety of factors, from organizational and social factors [15] to users’ characteristics (eg, user dexterity and anatomy and positive attitude) [16-19] or mobile features (eg, screen and buttons size, poor resolution, and usability) [6,20-22], would influence the successful adoption of mobile apps among physicians.

### Prior Work

Usability and quality issues have been reported as central to user engagement with mobile apps [6,21]. The research team previously developed a mobile app aimed at assisting physicians in prescribing head computed tomography (CT) scans based on appropriateness guidelines, the Head CT Scan Appropriateness Criteria (HAC) mobile app [1]. However, during that study, neurology and neurosurgery residents expressed concerns about usability issues despite their interest in using the app. Therefore, before proceeding with full implementation, it is essential to identify and address usability problems of the app using mixed methods that involve participation from final users.

### Goal

In this study, we seek to investigate the usability of HAC app using mixed methods research involving quantitative analysis of the Mobile Apps Rating Scale (MARS) for quality assessment, quantitative and qualitative think-aloud (TA) usability testing, and debriefing across 3 phases.

## Methods

### Study Setting

This study was conducted as part of a broader effort to develop a mobile app, known as the HAC app, based on clinical guidelines. The development occurred at an academic hospital with Kashan University of Medical Sciences (KAUMS) in Iran, which has 510 beds. This newly developed HAC app allows end users to search for appropriate CT scans based on diseases, signs, symptoms, and modalities, such as CT, CT angiography (CTA), and MRI. Appropriate CT scans refers to imaging studies that are deemed clinically justified and indicated based on established medical criteria, including patient symptoms, signs, and relevant clinical history, in accordance with evidence-based guidelines and best practices in diagnostic radiology.

The study involved 16 medical interns from an academic hospital at KAUMS. For this study, the focus was on assessing the end-user usability through a TA approach [23,24], evaluating the quality of the HAC app using the MARS [25], and conducting informal debriefing sessions to gather insights and opinions for the medical interns regarding the HAC app.

### Profile of the HAC App: HAC App Content and Functionality

**Figure 1:**
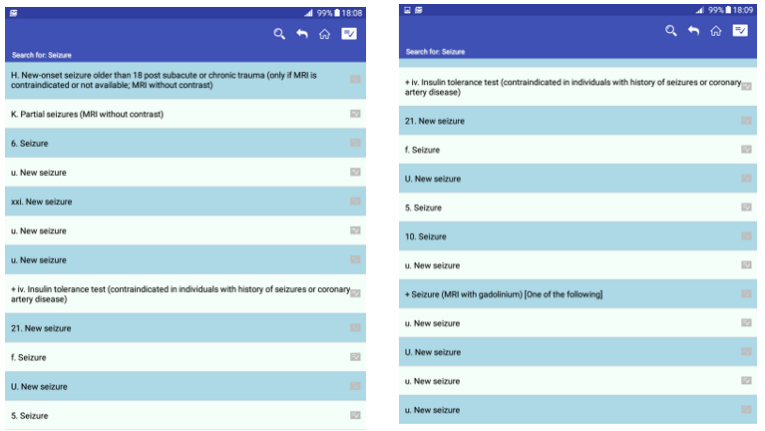
Head Computed Tomography Scan Appropriateness Criteria app search results for seizure.

**Figure 2:**
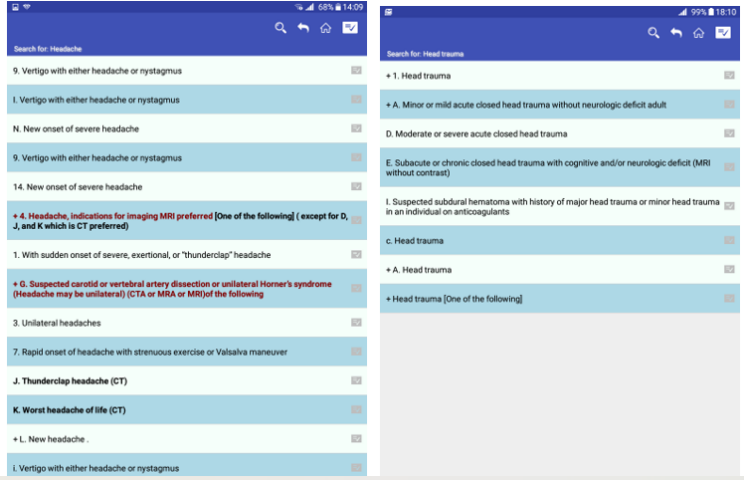
Head Computed Tomography Scan Appropriateness Criteria app search results for head trauma and headache.

The HAC app was developed using applied 4-tier architecture, including presentation, data service, business logic, and data access layers. The app was designed using JavaScript in such a way that allows it to be installed and be compatible with the latest version of Android in 2021 (version 12) as well as earlier versions. The HAC app encompasses essential criteria arranged by Care Core guidelines for head CT scans. Care Core provides a list of disease titles, for example, head trauma, which is supplemented by the list of clinical criteria in terms of signs and symptoms of the given disease. Clicking the plus sign (+) provides the detailed clinical criteria. Under each main heading or in front of each condition, the appropriate imaging procedure in MRI, CT, and CTA is provided. A shortlist menu is designed to organize and quickly find frequently used diseases or clinical criteria. It enables users to add common diagnoses to the shortlist menu. Screenshots of the functionalities of the HAC app are presented in Figures 1 and 2.

### Approaches to Conduct the Study

Three approaches have been used to conduct the research and achieve the study objectives.

### The TA Usability Testing

In this phase, we tested the HAC app’s effectiveness, efficiency, error, and learnability. The study objectives have been determined to ensure the accurate fulfillment of the tasks, the correct selection of the icons and buttons, and end users’ use of the mobile app without errors in an efficient way. The TA approach set out to determine the following measures to achieve the objectives:

- The effectiveness of participants’ navigation of the app was measured by the accurateness and completeness of the HAC app on CT scan ordering based on diseases, signs, symptoms, and modalities, for example, CT, CTA, and MRI.
- The efficiency of the participants was specified by the number of touch targets on the app screen and the task completion time.
- The simplicity and learnability of the HAC app were measured by the number of tasks that were easily completed and the severity of errors made by the users.
- Errors indicated the number of user mistakes when using the HAC app.

### MARS Quality Assessment

To evaluate the HAC app quality in terms of engagement, functionality, Aesthetics, information, and subjective quality, we applied the MARS tool [25], and the following dimensions were addressed:

- the overall quality score of the HAC app and its subscales, including Engagement, Functionality, Aesthetics, Information, and Subjective Quality
- a statistically significant difference between MARS subscales quality score
- a statistically significant difference between 2 sets of pairs of MARS subscales (eg, Engagement and Functionality or Functionality and Aesthetics)
- the correlation between MARS subscales for the HAC app
- the significant relationship between medical interns’ characteristics (ie, age, gender, and interest in using mobile apps for learning and clinical practice) with MARS subscales

### Debriefing

An informal debrief was conducted to review and digest interns’ general ideas about using mobile apps and physicians’ expectations for a suitable mobile app. It was also applied to collect underexplored facts for further revision of the HAC app.

### Study Design and Data Analysis

Our study design was primarily based on methodological triangulation through the use of mixed methods research, and investigator triangulation to enhance the understanding and interpreting the results [26].

The mixed methods study involved quantitative (MARS quality assessment and TA quantitative usability testing) and qualitative methods (TA qualitative usability testing and debriefing) across 3 phases. By using the technique of investigator triangulation, a variety of researchers, such as medical practitioners, experts in health information technology, and professionals in health information management, were involved in the gathering and analyzing of the data. The details of each phase will be discussed in the subsequent sections.

### Phase 1: TA Usability Testing Approach

#### Design

The study used a TA study design to explore the user’s cognition, including feelings, thoughts, and whatever else comes to mind while interacting with a system to perform a task. This standard data collection method for assessing users’ cognitive behavior during system interaction helps identify errors and necessary changes [23,24].

There are 2 fundamental usability testing methods: qualitative and quantitative [27]. Qualitative methods primarily aim to explore users’ interaction experiences with a product and describe possible issues they encounter [28]. In contrast, the quantitative methods use various metrics, such as task times, completion rates, and errors, to measure and categorize the errors and problems users encounter during usability testing [29]. Both qualitative and quantitative methods were applied in this study to reach the research objectives. Usability evaluation was also conducted in the different stages of a product development life cycle. Formative evaluation was done in the early product development life cycle to shape the design direction. Summative assessment was performed toward the end of the product development (final product) to evaluate its performance against a set of metrics (eg, time on task and success rate) [28]. The participants implemented summative usability testing in this study to evaluate the performance of the HAC app.

#### Participant Recruitment

Previous evidence confirms that about 5 to 15 participants are sufficient to perform TA to enhance the expected level of problem discovery [30]. We recruited 16 medical interns who participated in 3 phases of the study. We applied social media to attract medical interns to join the study. We posted our research profile on the medical students’ academic and social media channels, including the study title, research team, and overall study objectives. We invited those who finished their clinical internship in emergency medicine to participate in this study. Our multidisciplinary research team, including clinicians, significantly streamlined the recruitment process. The research team, consisting of members from diverse disciplines, encouraged their previous students to participate in the research. No rewards or compensations were paid to the participants.

#### Protocol

The TA usability testing was conducted in multiple sessions of the same activity. Developing a study protocol to ensure the consistency of each activity in each session helped the facilitator give all the necessary information to the participants, which seemed imperative [28]. The study protocol in this study consisted of session introduction, information capture methods (ie, observation and videotaping), task scenarios, user interactions with the product and any identified product problems and difficulties, and measurement criteria, which are discussed in the following sections.

#### Session Introduction

Interested volunteers were contacted to schedule face-to-face visits. TA sessions were held in the physicians’ actual workplace. It is widely believed that evaluations conducted in the field resemble the set-up that matches the user’s real work context, providing “ecological validity” to the study and accurately reflecting the users’ context [28].

Once the researcher arrived in the field, they gave the participants an overview of the session and the overall goals. They let them know about the presence of any facilitator or observers in the session and the rules for conducting the usability testing.

### Ethical Considerations

#### Overview

This study was approved by the ethics review board at KAUMS (code #IR.KAUMS.MEDNT.REC.1399.075). The participants were informed the same and emphasized the voluntary nature of participation, assuring them of the confidentiality of information. The nonevaluative environment of the TA session was also explained by a trained moderator (researcher). Then, participants who attended the face-to-face meeting consented to participate in the study and TA session run.

#### Data Collection

The usability data collection protocol was generally implemented via 2 approaches: concurrent TA and retrospective TA protocols [31].

Because concurrent TA is more objective and less dependent on users’ memory and prior experience of completed tasks compared with retrospective TA, concurrent TA was adopted as a standardized method to conduct usability testing of the HAC app [28,32].

Considering that most users are uncomfortable installing something on their devices (eg, mobiles or computers) [28] and the importance of using the same tool to capture usability-testing data, interaction with the HAC app was done via an Android mobile phone dedicated only to the research purposes. A portfolio of methods, including video screen-recording software, audio and screen recording, and notetaking, were applied to collect data. Four scenarios containing 4 to 6 tasks were given to the participant to interact with the HAC app. All the activities to accomplish the scenario, including the number of touch targets on the app screen, the task completion time, and the elapsed time, were captured using the free AZ Screen Recorder for Android (AZ Screen Recorder) [33].

Three evaluators facilitated the testing sessions and analyzed the results. The researchers adopted the verbal protocol to collect the data. Although the verbal protocol is the most traditional protocol with limited probing methods compared with active users’ participation methods, such as communication‐based and coaching protocols, it resembles an authentic context experience by not offering any external assistance to the users [34].

Thus, 1 researcher supervised the evaluation session, but neither user received instruction during the task performance stage. Attention was given to shortening the testing process and keeping a participant on the phone for >10 minutes [28]. Each TA session lasted for about 20 to 30 minutes.

#### Tasks Scenario

The scenarios (including their goals and actions) were designed to examine different parts and functions of the HAC app and covered the most common tasks that a clinician may use in a typical working application. Usability problems were detected by researchers from analyses of user behavior and expressions during interactions with the system.

#### Measurement

A coding framework was developed according to 5 usability characteristics and based on the International Organization for Standardization and Nielsen’s definitions to recognize the specific user-computer interaction problems in detail to define the measurement criteria [35-37]. Nielsen put forward 5 usability attributes: learnability, efficiency, memorability, errors, and satisfaction [37].

Combining International Organization for Standardization and Nielsen usability attributes yields the following 6 criteria: efficiency, effectiveness, learnability, memorability, errors, and satisfaction. Because the participants only used the HAC app in this study, and there was no need to remember the options for the next session, we omitted memorability in our evaluation. The remaining 5 attributes were integrated into our coding framework [23].

We used the TA method to measure effectiveness, learnability, errors, and efficiency characteristics, and the MARS questionnaire was used to measure satisfaction.

Errors or usability problems were detected based on the analysis of the “critical issues” encountered by the participants during the interactions detected from the video reviews. Critical issues were defined as those that prevented task completion, “severe issues” were defined as those issues that caused significant slowdown or frustration, and “cosmetic issues” were the ones that remained and had minimal effect [38].

Learnability was evaluated by measuring the number of quickly completed tasks.

### Data Analysis

#### Phase 1: TA Usability Testing

##### TA Quantitative Part

Data analysis and measurements of usability metrics were addressed based on a coding framework mentioned in the study design and protocol section. The usability characteristics and problems and their severity rating are described as follows:

Efficiency was measured by two metrics: (1) the number of touches targeted and (2) the task completion time. The mean time taken for the users to perform each task was based on the following equation:

Efficiency = [(total of full completion of a task (1) or noncompletion (0) / (time spent on a task)] / [(total number of tasks × number of users)] × 100 **(1)**

Effectiveness was measured by the number of completed tasks (ie, task completion rate), indicating the task’s success rate. The extent to which the user can fully and accurately achieve their task goals. Effectiveness was measured using the following equation.

Effectiveness = [(number of successfully completed tasks) / (total number of tasks performed)] × 100 **(2)**

The range of effectiveness was taken as “awful” (0%-50%), “bad” (50%-75%), “normal” (75%-90%), and “good” (90%-100%) [24].

Learnability was evaluated by measuring the number of quickly completed tasks.

Errors were identified as the number of user mistakes when performing the tasks.

Satisfaction was measured based on the user’s total score on the MARS questionnaire.

##### TA Qualitative Part

The video reactions of the participants were transcribed verbatim. Usability data, characterized by users’ comments, silences, repeated actions, and error messages, were collected through the recordings. Three members of the research team analyzed the obtained content. Transcripts and usability problems were also reviewed to identify the most common concerns. In any case of discrepancy in content analysis, a third-party reviewer was consulted.

These differences were categorized based on the tasks in the scenarios (ie, measurements, zoom and magnifying, and contrast and window level).

Data collected during the TA tasks (phase 1) were analyzed using fundamental inductive content analysis consisting of data reduction, data grouping, and the formation of concepts to answer research questions [39].

The inductive process is a bottom-up process that looks at all the issues as a whole by aggregating similar issues together until all the issues have been sorted into groups. Once all the groups (ie, subcategories) had been sorted, they were labeled to create more significant categories [40,41]. Thus, at the end of this process, we identified significant usability category issues and the specific problems associated with each one.

#### Phase 2: Evaluation of the Quality of the HAC App Using the MARS Questionnaire

##### Design

The participants (16 medical interns) were asked to complete the MARS questionnaire immediately after the TA session. MARS is the most popular scale and a highly reliable tool designed to assist researchers, professionals, and clinicians in classifying and assessing the quality of mHealth apps [25].

##### Data Collection

A validated and reliable Persian language version of MARS was used to collect the HAC app quality data [42].

MARS consists of 23 items in 5 objective quality subscales:

- Engagement encompasses 5 items and mainly focuses on entertainment and interest features of mobile apps.
- Functionality includes 4 items and addresses the ease of use and functional capabilities of mobile apps.
- Aesthetics consist of 3 items and discuss mobile app layout and visual appeal.
- The information includes 7 items and mainly considers quality, quantity, credibility, and visual enhancement of included information.
- The Subjective Quality subscale of MARS focuses on the overall rating of the app, its benefits, and its value.

##### Data Analysis

Each subscale item was rated a 5-point score from 1 (inadequate) to 5 (excellent). Usually, the mean score and SD were used to rate the quality of apps. Since the number of items in each subscale was different, we also used this formula [(mean of subscale/number of items in subscale)×(100)] to compute the score out of 100 and compare the subscales. To calculate the total HAC app score, the [(total mean of HAC app/total MARS items×(100)].

Friedman test was applied to compare the users’ scores in 5 MARS subscales. The Wilcoxon test investigated the mean difference between 2 sets of pairs of MARS subscales. Spearman rank correlation coefficient was used to analyze the positive correlation between MARS subscales. Kruskal-Wallis and 1-way ANOVA tests were used to assess differences between medical interns’ characteristics and MARS’ subscales. All statistical analyses were performed using SPSS (version 16.0; IBM Inc) at a significance level of .05.

We applied inductive content analysis consisting of data reduction, data grouping, and the formation of concepts to analyze TA qualitative data and transform physicians’ ideas into categories in the debriefing phase.

#### Phase 3: Debrief Participants

A debrief session is an informal conversation to collect users’ experiences and [43] any features of the app that they particularly like or dislike, how easy or difficult it is to use, and what they think about the content and design of the app was discussed in the debriefing session. The medical interns’ general opinion regarding the effective mobile apps to assist HCPs in education or clinical practice was also investigated in this phase. During the analysis of the recorded videos and voices, it came to our attention that the debrief sessions which were carried out with the participation of clinicians’ research team, were the most active and engaging ones. To analyze and present the debriefed data, a narrative analysis method was used [44].

## Results

### Outline

The findings of each phase will be presented under the same headings in the methods section, including TA quantitative*,* TA qualitative, MARS quality assessment, and then a debriefing session.

### Phase 1: TA Usability Testing

#### Overview

Table 1 illustrates the scenarios, goals, and actions needed to complete the tasks.

**Table 1:** Descriptions of the scenarios used in the usability testing.

Scenarios	Goals	Actions
1. A head trauma patient was admitted to the emergency department. Please check if the CT^a^ scan indicated a patient with “minor or mild acute closed head trauma without neurologic deficit adult.”	According to the guidelines, search for appropriate imaging procedures for a given diagnosis.	Selecting the search icon Typing the disease title in the search box Finding the head trauma from the query list Clicking on the plus button Check if the imaging procedure is recommended for the patient.
2. A patient was admitted to the emergency department with “new onset of seizures older than 18 following acute trauma.” Please select the appropriate imaging procedure for the case.	To use appropriate imaging procedures for seizures.	Opening the search query Typing the seizures into the search box Navigating between items in the search list Selecting the appropriate imaging procedure based on the patient’s symptoms
3. Headache and vertigo are common symptoms at the emergency department. Please add headache to the shortlist for forthcoming queries.	To apply a shortlist menu to collect appropriate imaging procedures for common diseases and symptoms.	Adding headache to shortlist menu Backing to the first page Opening the shortlist Deselecting the items you are not interested in anymore
4. A patient with proven subarachnoid hemorrhage (negative angiogram) was admitted to the hospital for follow-up. Please check for appropriate imaging procedures.	To use the CT^a^ or CTA^b^ button to access subarachnoid hemorrhage.	Opening list of diseases under the title of CT Finding subarachnoid hemorrhage Moving one step backward Selecting the CTA button Navigating between items in the search list Click on the plus sign to search for detailed information on subarachnoid hemorrhage and its subgroups.

^a^CT: computed tomography.

^b^CTA: computed tomography angiography.

#### TA Quantitative

#### Efficiency

On the basis of the equation 1, the HAC app’s relative overall efficiency was 97.8%. The average time spent for each scenario was 97.5 seconds, and the number of additional clicks was 0.93. The highest average of performing scenarios belonged to scenario 3 (109.25 seconds), and the lowest average was related to scenario 4 (83.875 seconds). Among the users, the highest total average time for 4 scenarios was related to user number 3 (161.8 seconds), and the lowest time was for user number 11 (58.0 seconds).

#### Effectiveness

The HAC app’s effectiveness in assisting users in performing the scenarios based on the equation 2 was good (97%). Of 16 users, 14 (88%) completed all 4 scenarios, 2 (13%) completed 3 scenarios, and 2 (13%) users had difficulty performing scenario 2, which was focused on searching for “new onset of seizures older than 18 following acute trauma.” The characteristics of this scenario that caused usability issues have been discussed under the heading TA qualitative, “inefficient data presentation and information control,” and “poor searching capabilities” (Figures 1 and 2).

#### Learnability

Out of 16 users, 11 (69%) managed to complete 4 scenarios, 4 (25%) users managed to complete 3 scenarios without encountering critical issues, and 2 (13%) users faced critical issues to complete 2 scenarios.

#### Errors

Out of 16 users, 10 (63%) users did not make any errors while doing the scenarios, and 6 (33%) users were able to do the scenarios with >1 errors (Table 2).

**Table 2:** Matrix of efficiency and effectiveness of the Head Computed Tomography Scan Appropriateness Criteria (HAC) mobile app^a^.

User number	Efficiency, s						Effectiveness
	Scenario 1	Scenario 2	Scenario 3	Scenario 4	Total average	Total scenarios completed	
1	98	189	150	210	161.8	3	
2	75	101	134	82	98.0	4	
3	192	135	129	108	141.0	4	
4	92	86	115	57	87.5	4	
5	74	125	63	84	86.5	4	
6	115	73	129	53	92.5	4	
7	115	83	82	52	83.0	4	
8	69	93	106	127	98.8	4	
9	60	109	89	80	84.5	4	
10	70	117	170	82	109.8	3	
11	34	57	79	62	58.0	4	
12	40	97	125	45	76.8	4	
13	109	132	87	64	98.0	4	
14	72	83	95	67	79.3	4	
15	101	185	95	107	122.0	4	
16	110	59	100	62	82.8	4	

^a^Average efficiencies: scenario 1=89.125, scenario 2=107.75, scenario 3=109.25, scenario 4=83.87, total average=97.5.

#### TA Qualitative

The results of the inductive content analysis regarding usability issues were grouped into 8 main categories and discussed below.

##### Lack of Finger-Friendly Touch Targets

Most participants had difficulty tapping the target buttons, such as the shortlist button, or icons, such as the plus sign (+) on the screen, and it was an intensive task to perform successfully. The participants stated that the given features are inappropriate for finger-touch targets. It might be due to the wrong size of the buttons or the need for more padding between buttons and icons around the edge of the screen. Consequently, it led to selecting the wrong part of the screen and frequent mistapping of the shortlist menu. Most participants often used this statement: “I cannot get the button.” Failure to press the targeted button and retouching the icons multiple times occurred frequently, resulting in a long time on the task and decreased efficiency. Moreover, it caused a failure of task completion by 2 users and reduced the effectiveness of the HAC app.

##### Poor Search Capabilities

Navigating the diseases and signs and symptoms was case-sensitive to the upper case. It made the searching diagnosis and signs and symptoms keywords awkward. The participant struggled to find diseases and signs and symptoms that had not been typed in upper case. Some participants forgot the “case-sensitive” feature every time they started the new scenario. Thus, participants backed out and jumped over the navigation process or tried to find the given case from a long list of search results. Both situations made it time-consuming and inefficient and caused participant frustration.

##### Input Problems

The main complaint by the participants was that the font size was inappropriately amplified with the limited mobile size. Participants mentioned that typing on the mobile phone screen was an intensive task. We found some difficulty in typing on the small screen; all the participant’s attention was focused on what they had typed. The “case-sensitive” feature in searching data amplified the problem. The lack of finger-friendly touch targets also made the typing more cognitive load and distracted from their main concerns, interacting with the patients*.*

##### Inefficient Data Presentation and Information Control

Another usability issue that caused frustration among users was inefficient data presentation and information control. To apply the HAC app, users entered specific diseases, signs, or symptoms enclosed in the Care Core guideline in the “Index” box. However, the list of clinical criteria under the disease heading was grouped using the plus sign (+) to provide a proper data presentation. A long list of conditions in the form of a dropdown menu enclosing the common signs and symptoms made it confusing for the participant. Since the mobile screen was too small, providing a long list of search results makes it time-consuming and inefficient. The lack of proper information layering and data categorization made it difficult for the participant to scroll the list. The participant commented the following:

It requires much attention and is very inconvenient since we need to interact with patients, other colleagues, and clinical settings environment.

The critical issue was related to bringing cognitive load to the participants.

##### Unclear Control and Confirmation

Another failure dealt with providing feedback and confirmation. The participants expected the HAC app to inform them about what was happening, using appropriate feedback. For instance, when they were asked to add a given disease to the shortlist, they waited for a dialogue to let them know the conditions were added. The absence of the appropriate feedback resulted in the users being moved to the shortlist and checked on if the command was run. The exact process occurred when they were asked to remove the given disease from the shortlist. The users awaited a confirmation dialogue regarding spoken questions, such as a “yes” or a “no,” to remove the disease from the shortlist before executing the removing command. Without a physical response, users did not know the current system status and were not confident about the consequences of their prior actions. They felt confusion and frustration.

##### Lack of Predictive Capabilities

Some participants expected more predictive capabilities and automation to optimize manual tasks and increase efficiency across various functions. For example, a participant stated the following:

We prefer the HAC app to automatically move the most visited diseases or signs and symptoms to the shortlist menu.

They believed the sole manually supported feature for making a shortlist menu could be more efficient and less time-consuming.

##### Poor Assistance and Support

The participants thought some features on the HAC app, such as shortlists highlighted in red or items with the plus sign, were difficult to recall or interpret and caused cognitive load. The participants needed assistance or information to learn more about these features, such as tooltips, which display informative text, such as a description of its function when users hover over, focus on, or tap an icon. They were looking for a help tab and found it unclear because it was at the bottom of the “About us” tab. It caused the HAC app to be less self-descriptive and more dependent on external help, which needed to be clarified and made clearer.

##### Unclear Navigation Logic

Some fundamental navigation control issues (eg, “back” function) were also reported during usability testing. For example, the participants tended to click the back button to return to the previous page, but it actually led them back to the home page. This drawback can lead to work duplication and frustration in task completion.

### Phase 2: the Quality of the HAC App Using MARS

#### Analysis of Overall Quality Scores of the HAC App

**Figure 3:**
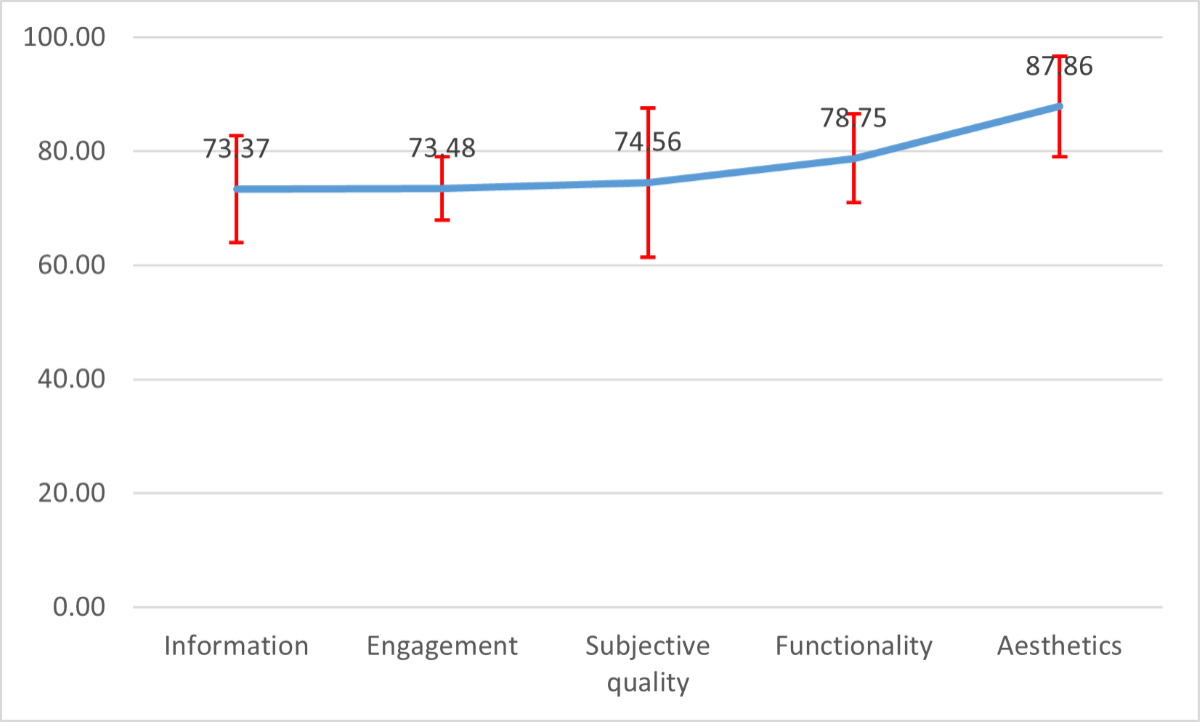
Overall quality scores of the Head Computed Tomography Scan Appropriateness Criteria app.

Table 1 indicates that the overall quality score of the HAC app was favorable (82/100). Among the 4 MARS subscales, Information (73.37/100), and Engagement (73.48/100) had the lowest scores while Aesthetics had the highest score (87.86/100; Figure 3).

#### Analysis of Significant Differences and Correlation Between MARS Subscales

Using the Friedman test, the users’ scores in 5 MARS subscales were compared, and the result revealed a significant difference (*P*<.001).

Wilcoxon test was applied to investigate the mean difference between 2 sets of pairs of MARS subscales. The results indicated a significant relationship between the Aesthetics subscale and Engagement (*P=*.001), Information (*P=*.003)*,* Subjective Quality (*P=*.004)*,* and Functionality (*P=*.02)*.* A significant relationship was also found between the Functionality and Information subscales (*P=*.01; Table 3).

**Table 3:** The mean differences between 2 sets of pairs of MARS^a^ subscale scores.

MARS subscales	Engagement score	Information score	Subjective quality score	Functionality score	Aesthetics score
Information score	0.909	—^b^	—	—	—
Subjective quality score	0.53	0.900	—	—	—
Functionality score	0.057	0.013	0.32	—	—
Aesthetics score	0.001	0.003	0.004	0.02	—

^a^MARS: Mobile Apps Rating Scale.

^b^Not applicable.

Spearman rank correlation coefficient presented a positive correlation between information with functionality subscales, *r*_.588_, *P*=.02. A positive correlation was also seen between information and satisfaction, *r*_.648_, *P*=.005. Table 4 indicates, in the subscale Information, the lowest score of the HAC app was “evidence base” (66.2/100), and the highest score was visual information (82/100). In the subscale Engagement, the lowest score of the HAC app was for “customization” (63.6/100), and the highest score was interest (90/100). In the subscale Functionality, the lowest score of the HAC app was for “performance” (67.4/100), and the highest score was “ease of use” (91.2/100). In the subscale Aesthetics, the lowest score of the HAC app was “visual appeal” (83.6/100), and the highest score was “graphics” (91.2/100).

**Table 4:** Head Computed Tomography Scan Appropriateness Criteria app scoring based on Mobile App Rating Scale 4 subscales.

			Scores, mean (SD)		Score out of 100
**Information**					
	Accuracy: the app contains what is described	3.6 (0.50)		72.4	
	Goals: specific, measurable, and achievable goals	3.6 (0.50)		72	
	Quality of information: the app correct, well-written, and relevant content to the goal	3.5 (0.63)		70	
	Quantity of information: the extent of coverage within the scope of the app	3.4 (0.72)		68	
	Visual information: visual (eg, charts, images, and videos) to describe concepts	4.1 (0.95)		82	
	Credibility: legitimate source of app	4.06 (0.25)		81.2	
	Evidence base: trialed and tested app	3.31 (1.07)		66.2	
**Engagement**					
	Entertainment	3.25 (0.44)		65	
	Interest: fun and entertaining of app	4.5 (0.63)		90	
	Customization: support all preferences for app features (eg, sound and content)	3.18 (0.54)		63.6	
	Interactivity: provide feedback, contain reminders, and notifications	3.8 (0.40)		76.3	
	Target group	3.62 (0.50)		72.4	
**Functionality**					
	Performance: accuracy and speed of the app functions and components (buttons and menus)	3.37 (0.80)		67.4	
	Ease of use: easy to learn how to use the app	4.56 (0.62)		91.2	
	Navigation: accurate, appropriate, uninterrupted moving between screens	3.8 (0.61)		76	
	Gestural design: consistency of (taps, swipes, and scrolls) across all components	3.9 (0.57)		78	
**Aesthetics**					
	Layout: arrangement and size of buttons, icons, menus, and content on the screen	4.43 (0.72)		88.6	
	Graphics: the quality and resolution of graphics used for buttons, icons, menus, and content	4.56 (0.51)		91.2	
	Visual appeal: look of app	4.18 (0.54)		83.6	

#### Medical Interns’ Characteristics and MARS Subscales

**Figure 4:**
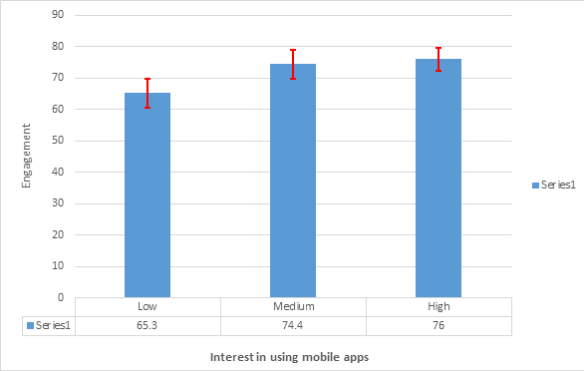
Significant difference between engagement and interest in using the mobile app.

Of the 16 users participating in the study, none had used the HAC app before, and only 1 (6%) person had used similar applications. Among them, 8 (50%) users believed using mobile apps for learning and clinical practice is helpful and were interested in using them. Figure 4 presents a significant difference between medical interns’ interest in using mobile apps for learning and clinical practice (low, medium, high) with the Engagement subscale using the Kruskal-Wallis test (*P*=.03).

**Figure 5:**
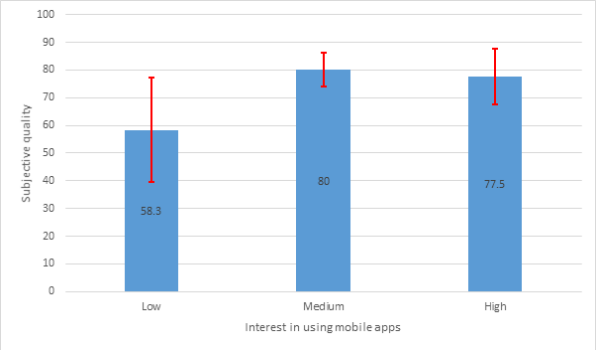
Significant difference between subjective quality and interest in using mobile app.

Figure 5 also indicates a significant difference between the medical interns’ interest in using mobile apps with subjective quality subscales using a 1-way ANOVA test (*P*=.04).

### Phase 3: Debrief

We explored how useful they perceived the app to be, any features they particularly liked or disliked, how easy or difficult it was to use, and what they thought about the content and design of the app, which was discussed in the debriefing session. Although all users appreciated the high simplicity and learnability of the HAC app, they debated that navigation between pages and search capabilities need serious consideration.

One of the participants wanted this tool to be equipped with voice recognition systems:

We use this tool while walking or moving in different parts of the hospital, and the possibility of typing or text entry increases the possibility of errors and, as a result, repeating the same action, which will reduce efficiency.

Participant 2

Another participant believed this tool should provide access to the app at different times and conditions:

I am a doctor, and my hands are bloody; I do not want to touch my mobile too much, and I prefer this app to be able to search for the proper CT scan based on voice.

Participant 15

Another participant expected that apps designed for students would pay more attention to the educational needs and learning styles of students:

I think this issue is so essential that medical education experts should also be used in the design of apps. Anyway, each of us has a style to learn. If this customization feature is not included in the design, surely some users will not be able to work with this system or at least feel comfortable and useful while working with it.

Participant 1

## Discussion

### Principal Findings

Our findings demonstrated that the HAC app was practical and had acceptable usability in efficiency and effectiveness. It also displayed a positive quality score based on the MARS scale. In contrast, results of the TA usability test revealed that the HAC app has 8 notable usability issues. The results proved that despite the willingness of researchers and the simplicity of quantitative and questionnaire-based approaches to conducting usability testing [6,45], the observational, TA usability testing provided more unbiased, trustworthy, and insightful data in describing mobile app usability.

Nevertheless, through data analysis of the MARS subscales also brought to light the HAC app’s usability issues, and its results support the qualitative TA results of this study. This agreement could be explained by the fact that MARS is a scale specifically designed to assess the quality of mobile apps [46]. Typically, the available usability scales and questionnaires are not highly reliable [6]; they are general scales designed primarily for evaluating the usability of computers or websites.

In addition, current usability and quality rating scales focus primarily on developers testing the usability of mobile apps, rather than end users who are patients or HCPs [46].

It is unlikely that usability issues will be thoroughly investigated in sole quantitative and questionnaire-based approaches [47] and need to be complemented by more objective and reliable approaches, such as TA methods.

In this study, HAC effectiveness assessment revealed that most users completed all 4 scenarios, although, 2 users faced problems completing scenario 2, which involved finding an appropriate imaging procedure for the “new onset of seizures” case. This failure may be due to the usability issues we categorized under “poor search capabilities” and “inefficient data presentation and information control.” As shown in Figure 1, “poor searching capabilities” and “poor data presentation” caused a long list of seizure conditions, confusing the participant. Since the mobile screen was too small, providing a long list of search results brought more cognitive load to select the correct item, and 2 participants were left to perform this scenario later. However, they never got back to the scenario again. Our results support previous research findings. In the study, Chen et al [48] introduced proper navigation and searching capabilities as significant factors for users’ rating of mHealth apps. Schwab and Langell [18] debated that ease of navigation is the foundation of an ideal mobile app since it smooths productivity and increases effectiveness. In the study to explore the usability of the physician-to-physician teleconsultation app in an orthopedic clinic, Choemprayong et al [49] presented mobile app usability issues in terms of data entry errors, presenting large-scale data and difficulty in selecting items from a list, which arise because of limited mobile screen size.

The HAC app also indicated acceptable efficiency and meantime completion for 4 scenarios. However, scenario 3 also showed the highest mean time completion. The problem might arise due to usability issues regarding the “lack of finger-friendly touch targets.” The limited screen size of mobile phones results in the inappropriate size of buttons or lack of enough padding between the shortlist button and icons around the edge of the screen. Our results agree with previous studies that tapping the mobile phone buttons correctly is a crucial factor; however, incorrect operations have been reported frequently in previous studies [49-51]. In addition to data presentation, the low resolution of smartphone screens can lead to data input errors [49]. Existing evidence revealed highly significant differences between user effectiveness and efficiency with button sizes. In the study, Conradi et al [22] reported substantial differences in error rate between button sizes (5×5 mm) compared with the other sizes (8×8 mm. 11×8 mm, and 14×14 mm. It has been debated that interaction with mobile devices due to limited screen size and resolution often requires additional considerations and a specially adapted interface. The literature also claimed that key size manipulation should be considered for users’ operation posture and activities (eg, standing, sitting, and walking) in mobile phone interactions [22]. However, the wide variation in optimal button size for mobile phones from 2.6 to 41.8 mm represents human–computer interaction in handheld devices. It is still in its infancy and requires more context-awareness to provide assistance based on the knowledge of its environment. Another possible explanation for the highest-time completion for scenario 3 is the usability issue categorized as “unclear control and confirmation” in this study. The participants of this study verbalized a lack of providing feedback on the HAC app when they were asked to add a given disease or sign and symptom to the list. The absence of the confirmation dialogue for successfully adding the given items to the shortlist resulted in the users moving to the shortlist and checking if the command was run. The exact process occurred when they were asked to remove the given disease from the shortlist. This rechecking caused work duplication and led to less efficiency. Work duplication has a significant and negative influence on physicians’ performance and has been introduced as physicians’ barrier to using mobile apps. In a study, Payne et al [52] found that physicians would use mobile apps to improve care workflow and productivity [38]. In another study, Ely et al [52] found that physicians believed if working with IT-related tools takes more than 2 minutes, they will not be efficient and practical for the point of care (39). Therefore, the effectiveness and efficacy of mobile apps serve as critical factors for physicians’ intention to use mobile apps [52,53].

Regarding efficiency measures, our results also indicated significant variation in scenarios’ time completion between the users. For example, user number 3 scored the highest total average time, nearly 3 times that of user number 11 (the lowest time), to perform the scenarios. Besides designing an optimal layout, significant variation in scenarios’ time completion between the users may be due to the user characteristics. Xiong et al [20] debated that touch accuracy in mobile phones requires proper motor skills and “hand dexterity” in the operating fingers. Schwab and Langell [18] and Ozkan Gokalp-Yavuz [16] also highlighted the importance of user anatomy (eg, average index or thumb fingertip size) and user dexterity (ie, motor skills) in users’ efficiency. Cho et al [51] reported usability problems related to the buttons of mobile apps developed using an eye-tracking system and retrospective TA usability evaluation.

The HAC app also showed a favorable quality score based on the MARS scale. However, the HAC app quality suffered from some drawbacks in Engagement and Information, which focus primarily on the effectiveness of apps in terms of interactivity, customizability, sending feedback, alerts, and reminders. Our results support previous results for assessing quality apps used by HCPs. In the study on drug reference apps in Taiwan, Chen et al [48] also reported poor engagement capabilities in terms of lack of entertainment, interactivity, and customization in the studied apps in Taiwan. In the study investigating influential factors in adopting a clinical photo documentation app for clinicians, Jacob discussed some drawbacks in engagement capabilities that need to be added for further revision of a given app [15]. Although few studies exist on using MARS to evaluate clinical apps adopted by HCPs, other relevant evidence supports our findings. In a qualitative study, Pokhrel et al [54] presented that HCPs prefer mobile apps that help them in their clinical practices, including “suggestive diagnosis and treatment after entering.” Reports of studies that focused on using other IT toolkits also revealed that the IT tool would be effective among HCPs if it would support interactivity, answer physicians’ questions, send feedback, and provide decision reasoning. Sandholzer et al [55] also introduced “prediction capabilities of mobile apps” as the most important preferences of medical students toward specific functionalities of future mobile apps. Despite the HAC app’s drawbacks in engagement and information subscales, its quality in aesthetics has shown favorable MARS scoring. In a study of preferences and perceptions of users regarding graphical user interface and user experience, Sandesara et al [56] reported that minimalist design improves user experience and user control to fulfill a task in a specific order and time. The author argued that “simplicity is the ultimate sophistication” [56]. To the best of our knowledge, no study has evaluated and reported the items of each subscale of MARS and research is lacking on the evaluation of adopting and usability testing of a mobile app by HCPs [1]. Lack of related literature to assess the items of each subscales of MARS led to poor in-depth understandings and meaningful perception of apps’ quality features in previous evidence. Therefore, it was impossible to compare HAC app quality rating with previous research properly. However, the results of quality assessment using MARS supports TA qualitative findings of this study. HAC app quality scoring in the functionality subscale revealed the minimum score belonged to the performance items, which focuses on the accuracy and speed of the app functions and components such as buttons or menus. Navigation also scored the minimum rating in the given subscale. In the subscale Engagement, the item customization that supports providing all necessary settings for apps features and the item interactivity that allows user input, providing feedback, and containing reminders and notifications also acquired the minimum scoring.

Our findings in the debrief session indicated that physicians with clear awareness and understanding of their clinical context and work processes tend to use other data input methods, such as voice recognition, to interact with the HAC app. The results of physicians’ workflow analysis and time and motion studies presented the medical profession as a multitasking job, not only managing patient care but also spending part of their activities on indirect tasks, from doing paper work and documentation to transitioning and traveling within the clinic area, or fetching or bringing something [57,58]. Thus, in designing mobile apps, performance accuracy and time on users’ tasks in different positions while walking or standing should be addressed appropriately. It has been argued that interaction with mobile devices while walking influences people’s visual acuity and suppresses this ability by nearly 20% compared with visual acuity while standing [22]. Conradi et al [22] debated that walking is prone to a very high number of error occurrences, which is remarkable in smaller buttons. Using mobile apps with text entry methods involves physicians experiencing various interaction issues in terms of difficulty in typing on the small screen, mistapping due to inappropriate size of the buttons or lack of spacing between buttons, poor data presentation, and so on. Any poor mobile interaction is attention-grabbing and makes physicians concentrate solely on interacting with the mobile app to increase their performance accuracy. It would distract them from their main concern, which is interacting with the patients.

Moreover, it results in a long time being on the task and decreases the efficiency and effectiveness of HCPs in clinical settings. Auditory and sonic interfaces occupy less visual attention and make users less engaged in the sole main task. Consequently, users can handle multiple tasks simultaneously [59]. Here, physicians should be equipped with an alternative input method, for example, speech recognition. Evidence revealed that speech recognition has the potential to be a more efficient and effective method to speed up the entry rates while declining the error rates. It was reported that speech recognition supports high entry rates (speaks at a mean entry rate of 13-45 words per minute while walking around) and a low error rate of <2% [60]. Given the requirement that medical interns suggested, they emphasized the importance of “context-awareness” design in mobile apps that focuses on capturing and exploring context-based information to describe any entity (eg, persons, places, objects, and workflows) embedded in the environment to fully understand and characterize users’ tasks [59].

### Implications

The evaluation framework used in this study can serve as a guide for the design and improvement of future clinical mobile apps to ensure they meet usability and quality standards for use by HCPs. Identifying usability issues through user feedback and analysis can help developers improve the usability and user satisfaction of clinical mobile apps among HCPs. Moreover, the results of the study can serve as a reference for HCPs and developers in selecting and implementing clinical mobile apps with acceptable usability and quality. It emphasizes the importance of multidisciplinary research, incorporating medical education specialists’ expertise, and considering user characteristics like motor skills and hand dexterity. The mixed methods approach used in the study, including MARS and TA analysis, can be adopted to gather valuable insights into user behavior and inform the design process of future apps for HCPs and developers. The study also suggests context-awareness design as a critical factor in developing meaningful IT–based solutions such as mobile apps.

### Limitations

However, our investigation is subject to some limitations. It was conducted using a limited sample a specific target group (medical interns), and attending physicians and residents were not involved in the study. No contributions from IT experts and app developers were included in the evaluation of the HAC app. The study focused on the usability and quality of the HAC app in a specific medical context in Iran, which may limit the applicability of the findings to other health care settings or countries.

### Conclusions

A mixed methods approach in evaluating the quality and usability of mobile apps yields valuable insights into the strengths and weaknesses of mobile apps. Adopting a holistic and multifaceted approach in evaluating mobile apps is highly recommended, as exclusively relying on a single methodology does not provide reliable and trustworthy information about the usability and quality of mobile apps. The results also presented that the unique characteristics of mobile devices, such as screen size, the users’ anatomical characteristics, and motor skills, influence users’ interaction and usability with mobile apps. Therefore, considering these characteristics and developing more tailored tools and methods for usability testing of mobile apps can bring potential benefits for developers, decision-makers, and HCPs.

## Abbreviations

CT: computed tomography
CTA: computed tomography angiography
HAC: Head Computed Tomography Scan Appropriateness Criteria
HCP: health care provider
KAUMS: Kashan University of Medical Sciences
MARS: Mobile Apps Rating Scale
mHealth: mobile health
TA: think-aloud
